# A new multi-criteria decision making method for the selection of construction contractors using interval valued fuzzy set

**DOI:** 10.1186/s13104-024-06769-w

**Published:** 2024-04-22

**Authors:** N. S. Nithya, Srinivasarao Thota, Laxmi Rathour, P. Shanmugasundaram

**Affiliations:** 1 Department of Software Systems, School of Computer Science and Engineering, Vellore Institute of Technology, Vellore, Tamil Nadu India; 2 Department of Mathematics, Amrita School of Physical Sciences, Amrita Vishwa Vidyapeetham, Amaravati, Andhra Pradesh 522503 India; 3 Department of Mathematics, National Institute of Technology, Chaltlang, Aizawl, 796012 Mizoram India; 4 Department of Mathematics, College of Natural and Computational Science, Mizan-Tepi University, Tepi, Ethiopia

**Keywords:** Contractor selection, Multi criteria decision making, Construction project, Interval valued fuzzy set, Digital weights

## Abstract

**Objective:**

This article introduces a novel approach called Digital Weighted Multi Criteria Decision Making (DWMCDM) that employs interval valued fuzzy sets to select the best contractor for building projects. The contractor is chosen based on the pre-qualification and bid evaluation phases. In the first phase, the distance between the actual and required skills of the significant criteria is determined, and it is then converted into digital weighted distances to identify the maximum number of criteria related to the specific project of each contractor. The second step ranks the best contractor based on the bid price and digital weighted distances.

**Results:**

The suggested technique integrates the pre-qualification and bid review phases to address project award delays and other restrictions. Finally, a real-world application is addressed to demonstrate the applicability of the proposed approach to any type of interval valued fuzzy inputs.

## Introduction

To maintain the dignity, competitiveness and scarcity of the work, the contractors who quote the lowest bid price remain in business. The selection of the lowest bidder is one of the main reasons for project delivery problem, when the contractor faced with a shortage of work, they desperately quoted a lowest bid price simply to stay in business with the expectation of be compensated by complaints [1, 2]. It includes multiple performance assessment criteria, Project quality, inefficient labor, on-time project delivery, and other non-price related aspects are impacted by the higher bid value. As a result, the project assignment should involve a number of choices regarding things like cost, quality, and other aspects. The performance of a project could be jeopardized without a sufficient and precise approach of choosing the best suitable contractor [3]. Any construction project's key issue is the delay that develops throughout the project, which extends the project's timeline [4].

The mathematical criteria must be included in the tender specifications whenever it is necessary to translate price bids into scores for conjunction with technical qualities of the proposal, such as quality or customer preferences [5]. The requirements could be in opposition, as in the case of customers who desire both quality and affordability. The decision-making process is complicated by conflicting criteria [6]. In actuality, the contractor pre-qualification phase (examination of predetermined criteria) and the bid evaluation phase (determination of minimum bid price) are assigned to the project. The pre-qualification is then the topic of a thorough examination to determine the current status of each contractor's management, technical, and financial capabilities [4].

The invention of mathematical and computational tools to assist the arbitrary evaluation of the performance criterion for decision-making is the subject of the operations research subfield known as multiple-criteria decision making (MCDM) [7]. Making decision in contractor selection includes multiple performance assessment criteria, both qualitative and quantitative and it is the main issue in supply chain management system. Numerous methods and algorithms are described in the literature; a few of these include the Fuzzy Analytical Hierarchy Process (FAHP) [8], Data Envelopment Analysis (DEA), Multi-Attribution Decision Making (MADM), and Multi-Objective Decision Making (MODM), as well as the TOPSIS and VIKOR models, which are used for supplier selection [9], performance evaluation [10], and project management [11]. In general, a multiple criteria decision making (MCDM) process consists of a group of decision makers, a number of admissible alternatives, and a number of criteria. Various decision making algorithms are discussed in the literature, see [12–22], references therein.

The human preference model is unclear in many real-world situations, and decision-makers may be reluctant or unable to give precise numerical values to comparative judgments [9]. Fuzzy logic is built on natural language and functions similarly to how people think. To face the factors of uncertain and imprecise information in real life situation, many theories have been developed, e.g. fuzzy sets [17, 23], approximation theory [24–26] etc. Intuitionistic fuzzy sets (IFS) was introduced by [27] to assign non membership grades and it was studied by [28, 29]. Neutrosophic fuzzy set (NFS) was introduced in 1998 by [30] to assign incomplete, indeterminate, and inconsistent information.

In decision-making management, when historical data is either unavailable or insufficient, expert opinion is the only source of knowledge that may be used to make a decision [15]. Fuzzy sets cannot give adequate explanations as the evaluation criteria, decision makers, and personal judgments vary. In order to clear up the ambiguity in this process, fuzzy sets with interval values IVFS are added [12, 31]. Decision-makers express their thoughts using language scales, and with the aid of an algorithm, they are further translated into interval valued fuzzy numbers with the help of different membership functions. MCDM technique has several qualities, numerous criteria, and a number of decision-makers. Decision makers must aid in the evaluation of n-contractors and all contractors in relation to each criterion. Our proposed method identifies the best contractor from a set of n-contractors and all contractors must be evaluated against to each criterion with the help of decision makers.

## Methodology

### Criteria for contractor selection

The main source of criteria which includes the related sub-criteria for assessing the pre qualification status of construction companies [32] given in Table 1.

**Table 1:** Criteria for contractor selection

Main criteria		Sub-criteria
*S*_*1*_	Firm’s capacity	Current workload, equipment availability, size of organization, number and scale of completed projects
*S*_*2*_	Quality	Quality of material, quality management system, experience and ability of quality control unit, equipments in quality control unit
*S*_*3*_	Management	Knowledge and capability of project management system, flexibility, responsiveness, reputation
*S*_*4*_	Experience	Experience of labor, expertise in particular construction method, past performance, technical manpower
*S*_*5*_	Financial Stability	Financial status, past failures, claims dispute history, length of time in business
*S*_*6*_	Safety	Project control and risk management capacity, health and safety records, past safety performance

### Preliminaries

Definition 1: [23] A fuzzy set *A* is a universe of discourse *X* characterized by a membership function $\mu_{A} (x)$* → *[0, 1], where $\mu_{A} (x)$*,* ∀ *x* ∈ *X,* represents the degree of truth of *x* in fuzzy set *A*.

Definition 2: [7] Let *X* be an universal set with cardinality *n.* Let [0, 1] be the set of all closed sub intervals of the interval [0, 1] and elements of this set are denoted by uppercase letters. If *M* ∈ [0, 1], then it can be represented as *M* = [*M*_*L*_, *M*_*U*_], where *M*_*L*_ and *M*_*U*_ are the lower and upper limits of *M*. An Interval-Valued Fuzzy Set (IVFS) *A* in *X* is given by $A = \left\{ {\;(x_{i\;,} M_{A} (x_{i} ):x_{i} \in X\;} \right\}$ Where $M_{A} :\;X \to \;[0,\,\,1]$, *M*_*A*_* (x*_*i*_*)* denote the degree of membership of the element *x* to the set *A* and 0 ≤ *M*_*AL*_ ≤ *M*_*AU*_ ≤ 1.

#### Algorithm

In this section, we proposed the Digital Weighted Multi Criteria Decision Making (DWMCDM) for contractor selection using IVFS. Suppose *S* is a set of skills of contractor, *P* is a set of projects and *C* is a set of contractors. Let *Q: C *$\to$* S* is an interval valued fuzzy relation from the set contractors to the skills and *R: S *$\to$* P* is an interval valued fuzzy relation from the set of skills to the projects. The main steps of our proposed method are as follows:

- 1. Decision makers convey their opinions on the actual performance levels of the contractors' competencies using linguistic scales that are transformed into an interval-valued fuzzy membership functions.
- 2. Determine the necessary system assessment standards that are related to the scope of the projects.
- 3. Based on the project requirements, calculate the difference or deviation between the contractor criterions expected interval and observed interval.
- 4. Applying digital weights to each criterion will allow you to find the digital weighted distance score values.
- 5. Determine the digital weighted distance scores, which satisfies all required criteria’s, and confirm that all other values are 0.
- 6. Enter the price quotation matrix B for each project.
- 7. The bid values are set in accordance with the cost reduction criteria.
- 8. The value of the final score is equal to the corresponding product values of the above average weighted digital distance scores (in step-6) and the reciprocal of the project bid price.

### Steps involved in algorithm

The proposed method involves the following steps:

Step 1: The ratings of decision-makers contractors-skills relation matrix *Q* for each criterion in terms of the interval valued fuzzy set *(QL*_*ij*_*, QU*_*ij*_*),* where *i* = 1, 2, 3, …, *m* and *j* = 1, 2, 3, …, *n*. In order to find the actual and necessary gap between the skills of the significant construction projects criteria's, decision makers translate their judgments about the criterion expressed in terms of language scales into an interval-valued fuzzy membership function.

Step 2: Create an interval-valued fuzzy relation matrix *R* referred to the skills-projects relation matrix using the anticipated ratings of the decision-makers under each criterion*(RL*_*ij*_*, RU*_*ij*_*),* where *i* = 1, 2, 3, …, *m* and *j* = 1, 2, 3,…, *n*.

Step 3: The distance matrix (see Table 6) relations *D* = *[C*_*ik*_*]*_*mxp*_, where.

*C*_*ik*_ = *QU*_*ij*_*—RL*_*ji*_, *j* = 1, 2, 3, …, *n* and *i* = 1, 2, 3, …, *m*

Step 4: Applying the corresponding weights, the digital weighted distance matrix (see Table 7).

*W* = *[Z*_*ik*_*] *_*mxp,*_

where $$Z_{ik} = \left\{ {\begin{array}{*{20}c} {1000000\quad if\;0.01 \le {\text{C}}_{{{\text{ik}}}} \le 0.05} \\ {1100000\quad if\;0.06 \le {\text{C}}_{{{\text{ik}}}} \le 0.1,} \\ {1110000\quad if\;0.11 \le {\text{C}}_{{{\text{ik}}}} \le 0.15,} \\ {1111000\quad if\;0.16 \le {\text{C}}_{{{\text{ik}}}} \le 0.20,} \\ {1111100\quad if\;0.21 \le {\text{C}}_{{{\text{ik}}}} \le 0.25,} \\ \begin{gathered} 1111110\quad if\;0.26 \le {\text{C}}_{{{\text{ik}}}} \le 0.30 \hfill \\ 1111111\quad if\;0.31 \le {\text{C}}_{{{\text{ik}}}} \le 0.35 \hfill \\ \end{gathered} \\ {0\quad \quad otherwise} \\ \end{array} } \right.\;$$

Step 5: Calculate the sum of digital weighted distance score matrix (see Table 8) for each element.

$$S\, = \,\left[ {Sik} \right]_{mxp}$$

where $$S_{ik} = \sum\limits_{j = 1}^{n} {Z_{ij} }$$

Step 6: Identify and select the digital weighted distance scores, which satisfies all required criteria’s (see Table 9) and make it that all other values are zero.

Step 7: Enter the bid price matrix B (see Table 10) for the corresponding project.

Step 8: The reciprocal values (see Table 11) of the bid prices are selected in order to minimize cost criterion.

Step 9: Determine the final score matrix F (see Table 12) by multiplying the corresponding above average digital weighted distance score matrix and the reciprocal values of the bid prices.

## Application

Let us say there are six contractors. The universal set *C* = {*C*_*1*_*, C*_*2*_*,…,C*_*6*_} represents construction industry contractors with strong industrial backgrounds. The set *S* = {*S*_*1*_*, S*_*2*_*, S*_*3*_*, S*_*4*_*, S*_*5*_, *S*_*6*_} stand for firm capacity, material quality, management, experience, financial stability, and workplace safety respectively,. The set *P* = {*P*_*1*_*, P*_*2*_*, P*_*3*_} where *P*_*1*_*, P*_*2*_ and *P*_*3*_ stand for three types of residential buildings respectively. Interval valued fuzzy numbers and corresponding linguistic variables are listed in Table 2.

**Table 2:** Ratings of the interval valued fuzzy numbers and linguistic variable

linguistic variable	Very low (*VL*)	Low (*L*)	Medium (*M*)	High (*H*)	Very high (*VH*)
Fuzzy interval	(0,0.2)	(0.2,0.4)	(0.4,0.6)	(0.6,0.8)	(0.8, 1)

Step -1: Using the ratings of field experts for each contractor under each criterion in terms of linguistic scales are listed in Table 3, an interval valued fuzzy relation matrix *Q* called a contractors-skills relation matrix is determined. This matrix is then converted into an interval valued fuzzy set *(QL*_*ij*_*, QU*_*ij*_*).*The committee's findings are listed in Table 4.

**Table 3:** Ratings of the alternatives with regard to the criteria made by the decision makers

Alt	Decision makers (DM_1,_DM_2,_DM_3_) ratings for criteria *S*_*i*_					
	*S*_*1*_	*S*_*2*_	*S*_*3*_	*S*_*4*_	*S*_*5*_	*S*_*6*_
*C*_*1*_	*(M,* * M, H)*	*(M,M, M)*	*(L, M, H)*	*(M, H, M)*	*(H, H, M)*	*(VH, VH, H)*
*C*_*2*_	*(H, M, H)*	*(H,H, H)*	*(VH, H,VH)*	*(H, M, VH)*	*(M,VH, H)*	*(VH, VH, H)*
*C*_*3*_	*(M, M, M)*	*(M,H, H)*	*(VH,VH,VH)*	*(H, H, VH)*	*(VH, H, H)*	*(H, H, H)*
*C*_*4*_	*(H, H, VH)*	*(H,H, H)*	*(M,H, M)*	*(M, H, H)*	*(H, VH,H)*	*(H, M,H)*
*C*_*5*_	*(VH,VH,VH)*	*(H,M,VH)*	*(H, M, M)*	*(M, H,VH)*	*(M, H,H)*	*(VH, H,VH)*
*C*_*6*_	*(M, H,VH)*	*(H,M, H)*	*(VH, H,VH)*	*(H, M, H)*	*(M, H, M)*	*(H, VH, H)*

**Table 4:** Contractors vs skills matrix relation (using algorithm step-1)

*Q*	*S*_*1*_	*S*_*2*_	*S*_*3*_	*S*_*4*_	*S*_*5*_	*S*_*6*_
*C*_*1*_	(0.47,0.67)	(0.4,0.6)	(0.4,0.6)	(0.47,0.67)	(0.53,0.73)	(0.73,0.93)
*C*_*2*_	(0.53,0.73)	(0.6,0.8)	(0.73,0.93)	(0.6,0.8)	(0.53,0.73)	(0.73,0.93)
*C*_*3*_	(0.4,0.6)	(0.53,0.73)	(0.8,1)	(0.67,0.87)	(0.67,0.87)	(0.6,0.8)
*C*_*4*_	(0.67,0.87)	(0.6,0.8)	(0.47,0.67)	(0.53,0.73)	(0.67,0.87)	(0.53,0.73)
*C*_*5*_	(0.8,1)	(0.6,0.8)	(0.47,0.67)	(0.6,0.8)	(0.53,0.73)	(0.73,0.93)
*C*_*6*_	(0.6,0.8)	(0.53,0.73)	(0.73,0.93)	(0.53,0.73)	(0.47,0.67)	(0.67,0.87)

STEP 2: Construction of the interval valued fuzzy relation matrix *R*, which contains the field expert expected skills, where *R* may be either an expert matrix or any set of needed relation matrix. The recruiter's priority throughout the selection process for the project *Pi* may be on a selection of few skills, such as *S*_*2*_*, S*_*4*_*, S*_*5*_, and *S*_*6*_, whereas the other skills in the set *S* may not be a priority. As a result, each project *Pi'*s priority for the skills differs, and the field expert's recommendations are expressed in terms of an interval-valued fuzzy set *(RL*_*ij*_*, RU*_*ij*_*)* in Tables 4, 5, 6, 7, 8, 9, 10, 11, 12.

**Table 5:** Skills vs. Projects Relation (using algorithm step-2)

*R*	Residential type-I *P*_*1*_	Residential type-II *P*_*2*_	Residential type-III *P*_*3*_
Firm’s capacity (*S*_*1*_)	–	–	(0.8, 0.9)
Quality(*S*_*2*_)	(0.7, 0.8)	(0.7, 0.8)	(0.8, 0.9)
management(*S*_*3*_)	–	(0.8, 0.9)	–
Experience (*S*_*4*_)	(0.6, 0.7)	–	(0.7, 0.8)
Financial stability (*S*_*5*_)	(0.7, 0.8)	(0.7, 0.8)	–
Safety (*S*_*6*_)	(0.6, 0.7)	(0.6, 0.7)	(0.7, 0.8)

**Table 6:** Distance matrix *D* (using algorithm step-3)

*D*	*P*_*1*_	*P*_*2*_	*P*_*3*_
*C*_*1*_	(0.07, 0.03, 0.33)	(0.03, 0.33)	(0.23)
*C*_*2*_	(0.1, 0.2, 0.03, 0.33)	(0.1, 0.13, 0.03, 0.33)	(0.1, 0.23)
*C*_*3*_	(0.03, 0.27, 0.17, 0.2)	(0.03, 0.2, 0.17, 0.2)	(0.17, 0.1)
*C*_*4*_	(0.1, 0.13, 0.17, 0.13)	(0.1, 0.17, 0.13)	(0.07,0.03, 0.03)
*C*_*5*_	(0.2, 0.2, 0.03, 0.33)	(0.1, 0.03, 0.33)	(0.2, 0.1, 0.23)
*C*_*6*_	(0.03, 0.13, 0.27)	(0.03, 0.13, 0.27)	(0.03, 0.17)

**Table 7:** Distance matrix with digital weight W (using algorithm step- 4)

W	P_1_	P_2_	P_3_
C_1_	(11000000, 1000000, 1111111)	(1000000, 1111111)	(1111100)
C_2_	(1100000, 1111000, 1000000, 1,111,111)	(1100000, 1110000, 1000000, 1111111)	(1100000, 1111100)
C_3_	(100000, 1111110, 1111000, 1111000)	(1000000, 1111000, 1111000, 1111000)	(1111000, 1,100,000)
C_4_	(1100,000, 1110000, 1111000, 1110,000)	(1100000, 1111000, 1110000)	(1100000, 1000000, 1000000)
C_5_	(1100000, 1111000, 1000000, 1111111)	(1100000, 1000000, 1111111)	(1111000, 1100000, 1111100)
C_6_	(1000000, 1110000, 1111110)	(1000000, 1110000, 1111110)	(1000000, 1111000)

**Table 8:** Weighted distance matrix (using algorithm step-5)

S	P_1_	P_2_	P_3_
C_1_	3211111	2111111	2211100
C_2_	**4322111**	**4321111**	2211100
C_3_	**4333110**	**4333000**	2211000
C_4_	**4431000**	3322000	3100000
C_5_	**4322111**	3211111	3322100
C_6_	3221110	3221110	2111000

**Table 9:** Weighted distance matrix which satisfies all 4 criteria(using algorithm step-6)

S	P_1_	P_2_	P_3_
C_1_	0	0	0
C_2_	**4322111**	**4321111**	0
C_3_	**4333110**	**4333000**	0
C_4_	**4431000**	0	0
C_5_	**4322111**	0	0
C_6_	0	0	0

**Table 10:** Contractors bid price matrix B (in Millions) (using algorithm step-7)

	P_1_	P_2_	P_3_
C_1_	92	74	98
C_2_	**86**	**72**	89
C_3_	**87**	**73**	92
C_4_	**86**	70	96
C_5_	**85**	71	97
C_6_	89	68	95

**Table 11:** Reciprocal values of Contractors bid price matrix (using algorithm step8)

	P_1_	P_2_	P_3_
C_1_	0.010870	0.013513	0.010204
C_2_	**0.011627**	**0.013888**	0.011235
C_3_	**0.011494**	**0.013698**	0.010895
C_4_	**0.011627**	0.014285	0.010416
C_5_	**0.011764**	0.014084	0.010309
C_6_	0.011236	0.0140588	0.010526

**Table 12:** Final score matrix (F) for Assessment values (using algorithm step-9)

	Residential Type-I (P_1_)	Residential Type-II (P_2_)	Residential Type-III (P_3_)
C_1_	0	0	0
C_2_	50253.2	**60011.6**	0
C_3_	49804.8	59353.4	0
C_4_	**51519.2**	0	0
C_5_	50845.3	0	0
C_6_	0	0	0

From the above final score values (bold values), it is clear that the Residential Type-I projectP1, will be given to the contractor C4, Residential Type-II projectP2will be given to the contractor C2, and for Residential Type-III projectP3, no contactor satisfied all the four criteria.

## Results and discussion

In this paper, an IVFS-based Digital Weighted Multi Criteria Decision Making (DWMCDM) was proposed. It could identify the best contractor from a set of n-contractors in the construction industry contractor selection.First, the decision-makers express their thoughts about the skills of contractors using language scales, and with the aid of an algorithm for decision making, further translated into interval valued fuzzy sets and it was processed by the digital weighted operation, digital weighted fuzzy scores were generated in pre qualification stage. Then the second stage input the bid price values were set in accordance with the cost reduction criteria. Finally, ranks the best contractor based on the bid price and digital weighted distance scores using proposed algorithm for decision making.

## Conclusion

Our proposed DWMCDM method for contractor selection increases the performance of the construction industry and lowers the risk of project failure for the customer based on the following five factors (i) Choosing the project wise suitable criteria, rather than considering all criteria (ii) Digital weights helps to identify the best contractors based on number of criteria satisfied, rather than the highest possible scores by identifying the positive distance between each expected and actual interval. (iii) The contractor selection depends on scores in phase-I but not on lowest bid price (iv) Pre-qualification phase and the bid evaluation phase which will overcome the delay in project award and other constraints (v) Suitable for any kind of interval valued fuzzy sets. Moreover, our contractor selection method suggests that it can be used as a framework that is active for assessing actual contractor selection. We suggest that this study be expanded to include situations in which the problem's data has extensions from other interval valued fuzzy sets.

## Limitations

The length of the interval is quite small, and there is no direct measurement in any construction contractors in this study. All potential cases of fuzzy intervals are taken on assumptions. Due to time restrictions, this research is limited to initiatives involving the construction of buildings, but it is more broadly relevant. Decision experts of an industry are required to take for their opinion about the criteria affecting the contractor selection in such as construction system, contractor comparison, project allotment, etc., For simplicity, we evaluated a limited sample size and a small number of contractors for the case study; however, large sample can be taken with Maple implementation.

## Data Availability

This article contains all of the information that was created or examined throughout the investigation.

## Abbreviations

DWMCDM: Digital Weighted Multi Criteria Decision Making
MCDM: Multiple-criteria decision making
FAHP: Fuzzy Analytical Hierarchy Process
DEA: Data Envelopment Analysis
MADM: Multi-Attribution Decision Making
MODM: Multi-Objective Decision Making
IFS: Intuitionistic fuzzy Sets
NFS: Neutrosophic fuzzy Sets
